# Carglumic acid: an additional therapy in the treatment of organic acidurias with hyperammonemia?

**DOI:** 10.1186/1750-1172-3-2

**Published:** 2008-01-30

**Authors:** Virginie Levrat, Isabelle Forest, Alain Fouilhoux, Cécile Acquaviva, Christine Vianey-Saban, Nathalie Guffon

**Affiliations:** 1 Centre de référence Maladies Héréditaires du Métabolisme, Service de Pédiatrie, Hôpital Edouard Herriot, Lyon, France; 2 Service des Maladies héréditaires du métabolisme et dépistage néonatal, Centre de biologie et de pathologie Est, Lyon, France

## Abstract

**Background:**

Hyperammonemia in patients with methylmalonic aciduria (MMA) and propionic aciduria (PA) is caused by accumulation of propionyl-CoA which decreases the synthesis of N-acetyl-glutamate, the natural activator of carbamyl phosphate synthetase 1. A treatment approach with carglumic acid, the structural analogue of N-acetyl-glutamate, has been proposed to decrease high ammonia levels encountered in MMA and PA crises.

**Case presentation:**

We described two patients (one with MMA and one with PA) with hyperammonemia at diagnosis. Carglumic acid, when associated with standard treatment of organic acidurias, may be helpful in normalizing the ammonia level.

**Conclusion:**

Even though the usual treatment which decreases toxic metabolites remains the standard, carglumic acid could be helpful in lowering plasma ammonia levels over 400 micromol/L more rapidly.

## Background

### Hyperammonemia in organic acidurias

Organic acidurias such as methylmalonic aciduria (MMA) and propionic aciduria (PA) are autosomal recessive disorders of branched-chain amino acid metabolism. High ammonia levels are predominantly encountered in the first six months of life. Since the 1970's, hyperammonemia has been known to be due to an accumulation of propionyl-CoA which decreases the synthesis of N-acetyl-glutamate, the natural activator of carbamyl phosphate synthetase 1 [1].

A treatment approach with carglumic acid, the structural analogue of N-acetyl-glutamate, has been proposed to decrease high ammonia levels encountered in MMA and PA crises [2,3].

## Case presentation

We have reported 2 cases of first occurrence of PA and MMA with hyperammonemia who received standard treatment and carglumic acid in addition.

### Case 1

This patient, a girl born in June 1994, presented hypotonia, poor feeding, dehydration (20% weight loss) and low body temperature (35.5°) at 53 hours of age after a symptom-free interval. Laboratory studies showed metabolic acidosis (pH 7.1; bicarbonates 9 mmol/L) with massive ketonuria and hyperammonemia (427 micromol/L). Intravenous treatment was started with glucose (10 g/kg/d) and insulin (0.05 UI/kg/h), carnitine (400 mg/kg/d), sodium benzoate (300 mg/kg/d) associated with a hypercaloric protein-free diet (glucose 10 g/kg/d and lipids 3,5 g/kg/d the first 12 hours, then glucose 17 g/kg/d and lipids 5,5 g/kg/d) and vitamin therapy (hydroxocobalamin and thiamine). Carglumic acid was administered at 200 mg/kg/d during 48 h. Diagnosis of MMA was confirmed by urinary organic acid analysis (methylmalonic acid 20,000 mmol/mol creatinine).

**Figure 1:**
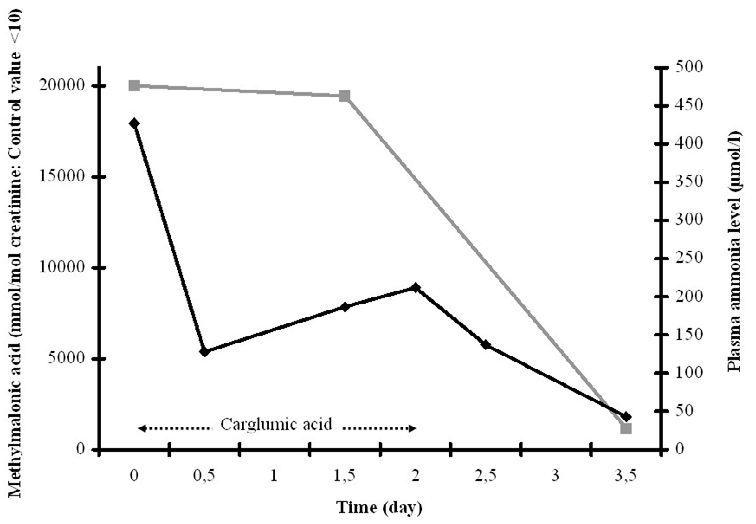
Plasma ammonia levels (black lines) and toxic metabolite excretions (grey lines) during the course of treatment: patient 1 with methylmalonic aciduria (MMA).

Ammonia decreased to 153 micromol/L within 12 h and normalized within 3.5 days, whereas methylmalonic acid excretion decreased more slowly (figure 1).

### Case 2

The second patient, a girl born in November 1997, presented hypotonia, seizures, poor feeding, dehydration, polypnea at 8 days of age after a symptom-free interval. Laboratory investigations revealed metabolic acidosis (pH 7; bicarbonates 3 mmol/L) with massive ketonuria and hyperammonemia (213 micromol/L). Intravenous treatment was started with glucose (9 g/kg/d) and carnitine (400 mg/kg/d) associated with hypercaloric protein-free diet (glucose 13 g/kg/d and lipids 3 g/kg/d the first 24 hours, then glucose 18 g/kg/d and lipids 4 g/kg/d). Acidosis decreased within 12 h but ketonuria remained and ammonia level reached to 526 micromol/L. Diagnosis of PA was confirmed by urinary organic acid analysis (methylcitrate 4.929 mmol/mol creatinine).

**Figure 2:**
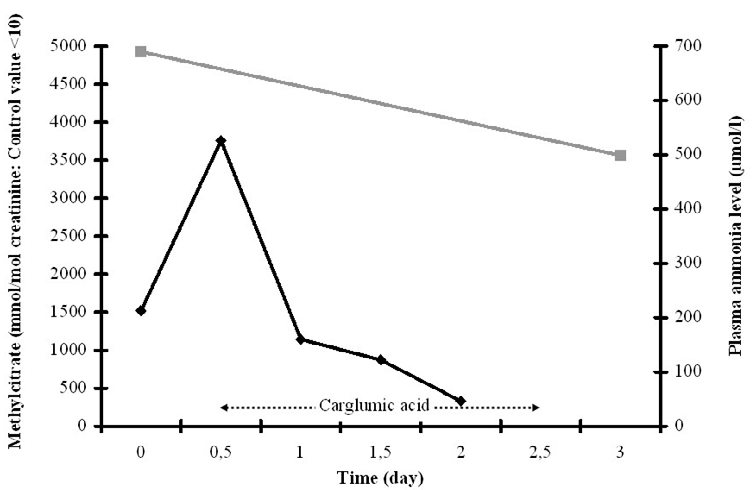
Plasma ammonia levels (black lines) and toxic metabolite excretions (grey lines) during the course of treatment: patient 2 with propionic aciduria (PA).

Carglumic acid was initially administered at 100 mg/kg followed with 70 mg/kg/d during 48 h. Ammonia was normalized within 36 h, whereas methylcitrate excretion remained elevated (figure 2).

## Conclusion

Neurological complications are frequently seen in patients with organic acidurias due to deleterious effect of toxic metabolites [4]. Therefore, the main aim of the treatment must be their removal by using standard treatment (high caloric, protein-free diet and carnitine). However, several studies have already demonstrated the direct toxic effect of ammonia on the central nervous system [5]. There is clearly an argument for enhancing ammonia removal by using additional therapy.

In patient 1, standard treatment and carglumic acid were started immediately at the time of diagnosis. The ammonia level decreased more rapidly than toxic metabolite excretion. In patient 2, the ammonia level increased after the beginning of standard treatment and decreased when carglumic acid was added. A strong correlation between serum propionate and blood ammonia concentration has been demonstrated mainly in the neonatal period [6]. In these two cases, the use of carglumic acid seemed to have a direct effect on ammonia removal, independently of the toxic metabolite excretions. Carglumic acid, with stimulating carbamyl phosphate synthetase 1, seems to be efficient more rapidly than standard treatment only. It enables to decrease ammonia level in the first 48 hours until standard treatment removes toxics metabolites. Consequently, non-experienced physicians should be aware that adding carglumic acid decreases ammonia level but does not indicate a decrease in accumulated toxic metabolites.

In organic aciduria patients with acute hyperammonemia, the clinical benefit of the use of carglumic acid has not yet been established. Based on our clinical experience, carglumic acid during 48 h seems helpful in lowering plasma ammonia levels over 400 micromol/L. Recently, three patients with MMA and PA have been treated for hyperammonemia with carglumic acid. In each case, carglumic acid appeared to accelerate ammonia detoxification [2,3]. Moreover, carglumic acid has been used in humans in the treatment of N-acetyl-glutamate-synthase deficiency with no major side effects [7].

Usual treatment remains the standard, however, carglumic acid in the treatment of hyperammonemia in organic acidurias seems to normalize ammonia level more rapidly. This effect should be assessed by a prospective multi-center study.

## Competing interests

The author(s) declare that they have no competing interests.
